# Supplementary figures and images for: Spread of endemic SARS-CoV-2 lineages in Russia before April 2021
Source: PLoS One. 2022 Jul 20;17(7):e0270717. doi: 10.1371/journal.pone.0270717 (PMC9299347; doi:10.1371/journal.pone.0270717)

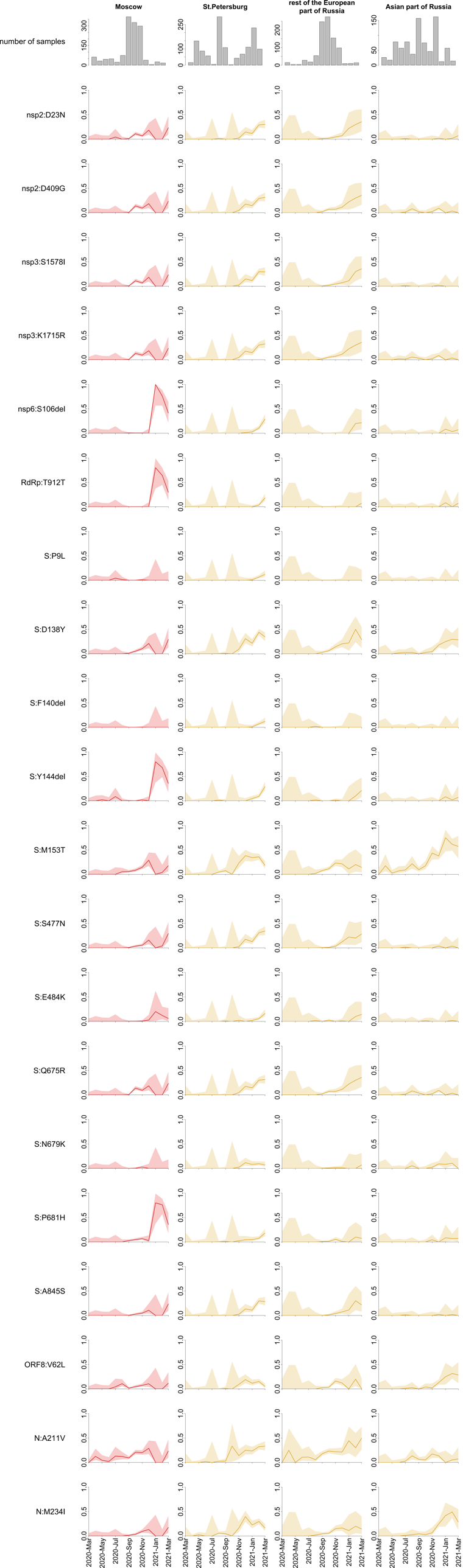

Supplement: S1 Fig — Notations are the same as in Fig 2. (PDF) [file pone.0270717.s001.pdf]

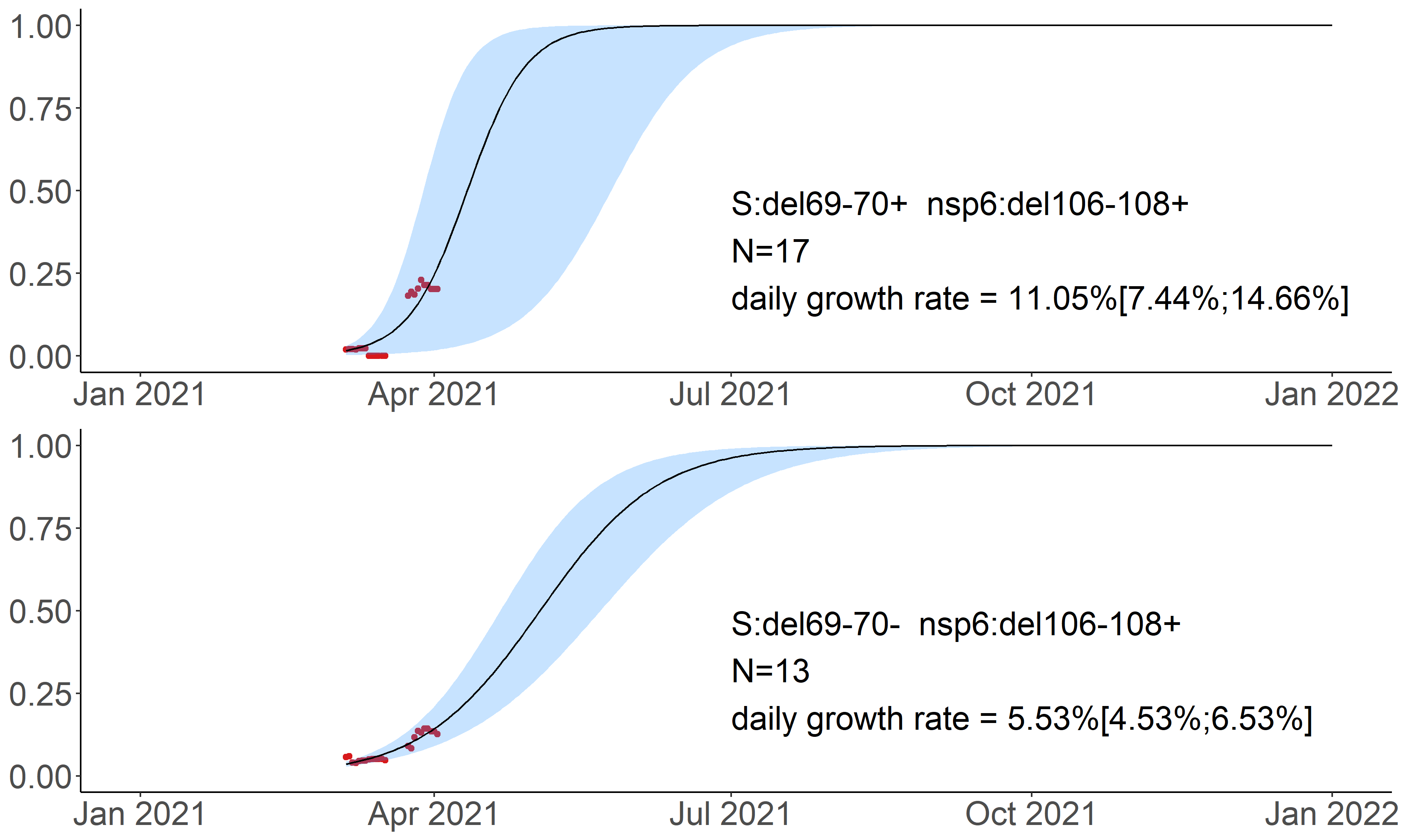

Supplement: S2 Fig — Notations are the same as in Fig 6. (PNG) [file pone.0270717.s002.png]

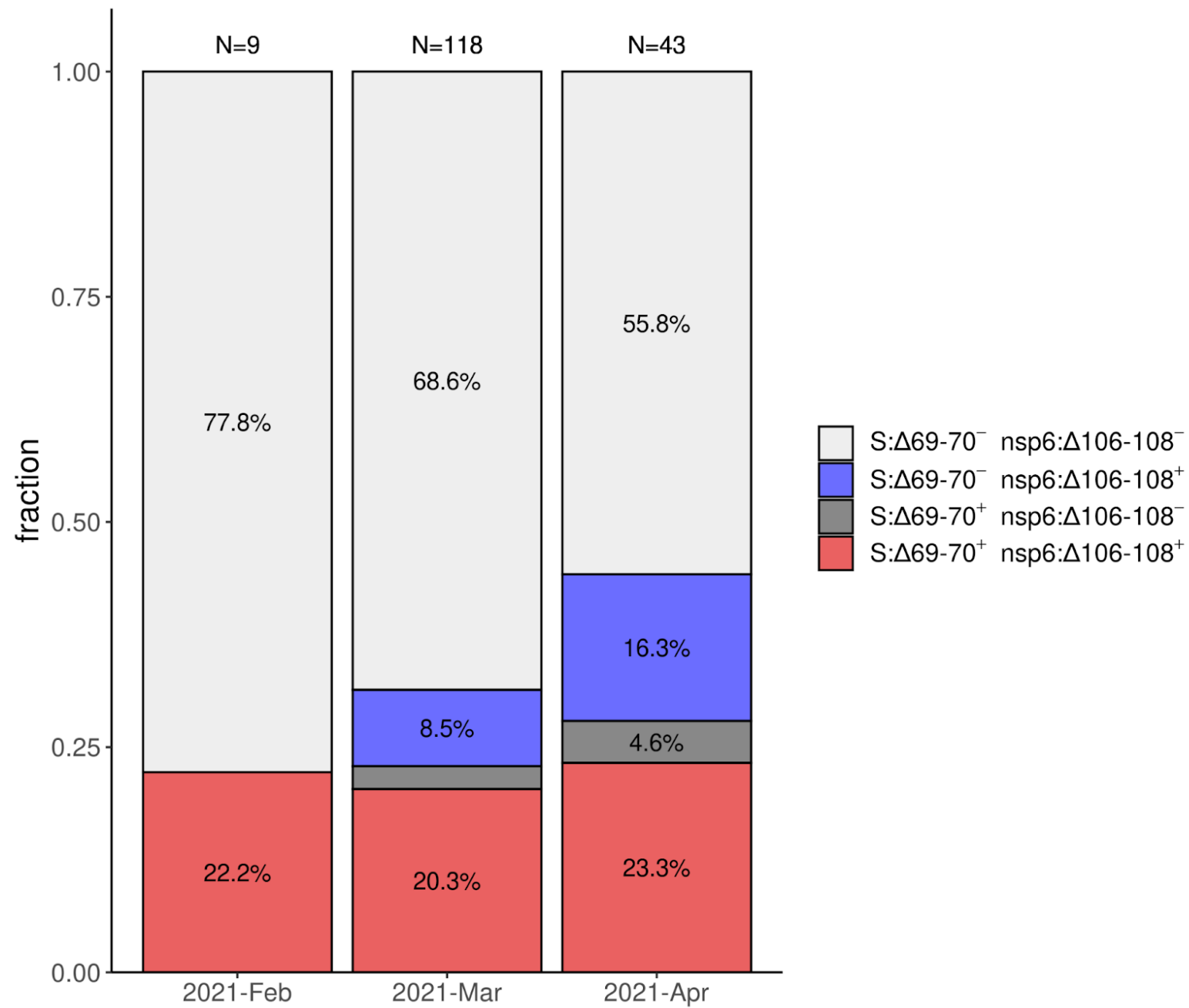

Supplement: S3 Fig — (PDF) [file pone.0270717.s003.pdf]

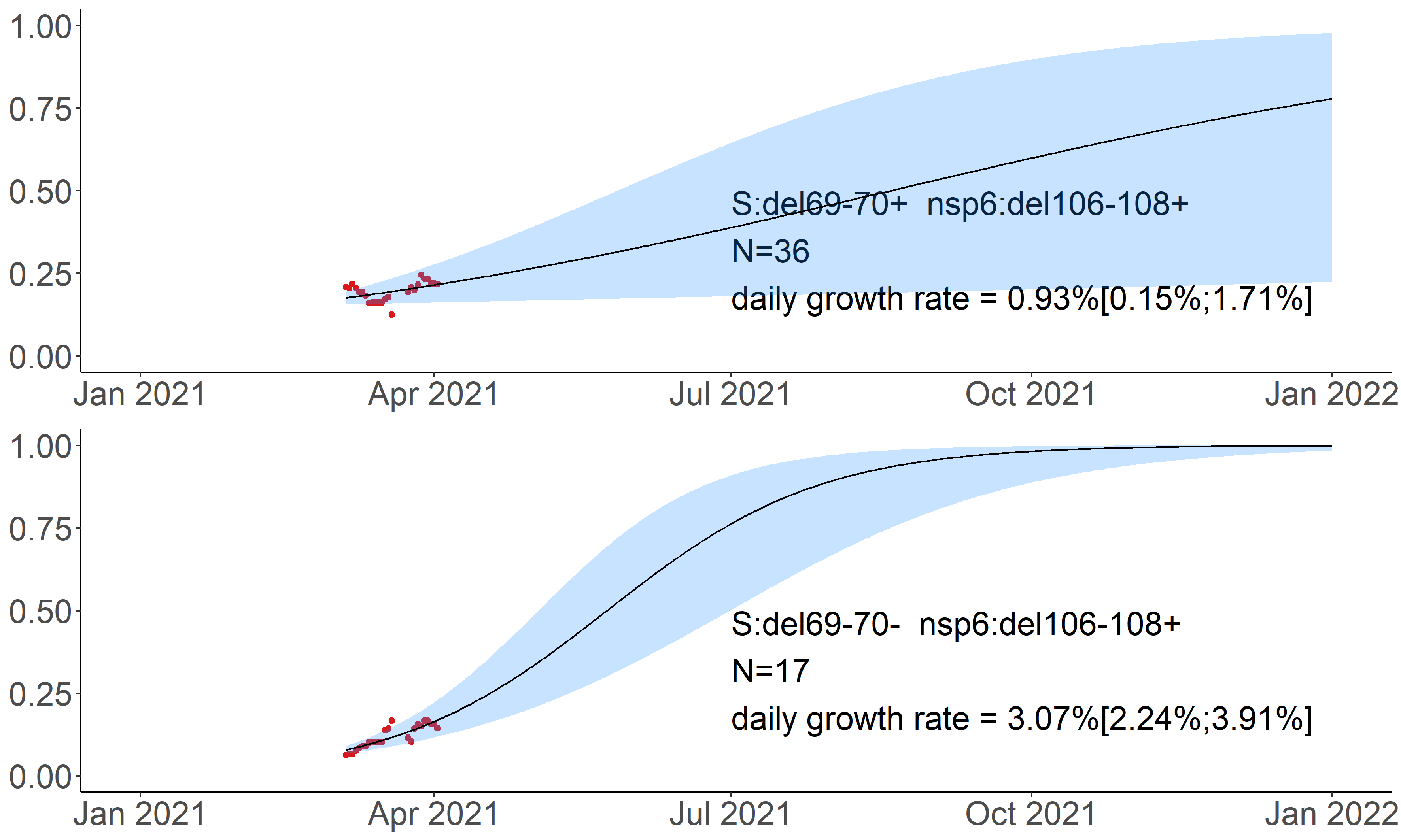

Supplement: S4 Fig — Notations are the same as in Fig 5. (PNG) [file pone.0270717.s004.png]
