# Supplementary material for: Spread of endemic SARS-CoV-2 lineages in Russia before April 2021
Source: PLoS One. 2022 Jul 20;17(7):e0270717. doi: 10.1371/journal.pone.0270717 (PMC9299347; doi:10.1371/journal.pone.0270717)
Supplement: S1 File — (PDF) [file pone.0270717.s007.pdf]

CoRGI consortium

Smorodintsev Research Institute of Influenza, WHO-recognized National Influenza Center of Russian Federation (Saint Petersburg, Russia)

Dmitry Lioznov

Daria Danilenko

Andrey Komissarov

Artem Fadeev

Anna Ivanova

Kseniya Komissarova

Mariia Sergeeva

Maria Pisareva

Veronica Eder

Tamila Musaeva

Maria Timofeeva

Andrey Ksenafontov

Nikita Yolshin

Petr Nekrasov

Helix LLC (Saint Petersburg, Russia)

Yury Andreichuk

Dmitry Denisov

Daria Sautkina

National Research Center for Epidemiology and Microbiology named after Honorary Academician N.F. Gamaleya (Moscow, Russia)

Alexander Gintsburg

Vladimir Gushchin

Inna Dolzhikova

Denis Logunov

Nadezhda Kuznetsova

Elena Shidlovskaya

Alexey Shchetinin

Maria Nikiforova

Andrei Siniavin

Andrey Pochtovyy

UMMC-Health LLC (Yekaterinburg, Russia)

Tatiana Platonova

Mikhail Sklyar

Elena Karbovnichaya

Artur Vorobyov

Eurasian Association of Therapists

Pirogov Russian National Research Medical University (Moscow, Russia)

Alexander Arutyunov

Privolzhsky Research Medical University (Nizhny Novgorod, Russia)

Ekaterina Tarlovskaya

City Clinical Hospital No. 7, Kazan, Tatarstan

Zulfia Kim

Petrozavodsk State University (Petrozavodsk, Russia)

Tatiana Kuznetsova

Krasnoyarsk State Medical University (Krasnoyarsk, Russia)

Marina Petrova

Privolzhsky Research Medical University (Nizhny Novgorod, Russia)

Alexandra Vaisberg

CORGI contributors (in alphabetical order)

Amur Regional Infectious Diseases Hospital (Blagoveshchensk, Russia)

Natalia Polovitsa

Astrakhan State Medical University (Astrakhan, Russia)

Olga Bashkina

Elena Vakalova

Oleg Rubalsky

Marina Samotrueva

Nikolay Kostenko

Botkin Memorial Hospital for Infectious Diseases (Saint Petersburg, Russia)

Denis Gusev

Maria Vashukova

Evgeny Venev

Centre for Hygiene and Epidemiology in Astrakhan Region (Astrakhan, Russia)

Elena Vokalova

Center for Hygiene and Epidemiology in Belgorod Region (Belgorod, Russia)

Liudmila Berdinskikh

City Hospital No 40 (Saint Petersburg, Russia)

Sergey Scherbak

Olga Shneider

Margarita Korzhanova

Gabrichevsky Moscow Research Institute for Epidemiology and Microbiology (Moscow, Russia)

Vladimir Aleshkin

Evgenii Rubalsky

Infectious Diseases Clinical Hospital №1 (Moscow, Russia)

Svetlana Smetanina

Natalia Antipyat

Marina Bazarova

Aleksandr Shagaev

Kamchatka Regional Children's Infectious Diseases Hospital (Petropavlovsk-Kamchatsky, Russia)

Svetlana Shapovalenko

Murmansk Regional Center for Specialized Medical Care (Murmansk, Russia)

Elena Labintseva

Office of the Federal Service for Surveillance on Consumer Rights Protection and Human Wellbeing in the Astrakhan Region (Astrakhan, Russia)

Natalia Nikeshina

Ogarev Mordovia State University (Saransk, Russia)

Irina Stepanenko

Omsk Research Institute of Natural Focal Infections (Omsk, Russia)

Valery Yakimenko

Ekaterina Gradoboeva

Aleksei Vasilenko

Elena Poleshchuk

Orenburg Regional Clinical Infectious Diseases Hospital (Orenburg, Russia)

Svetlana Soboleva

Denis Durchenkov

Irina Samoryadova

Peoples' Friendship University of Russia (RUDN University)(Moscow, Russia)

Olga Burgasova

Valeria Bacalin

Mikhail Odnoralov

Liubov Genralova

Republican Medical Genetics Center (Ufa, Bashkortostan, Russia)

Ildar Minniakhmetov

Rita Khusainova

Saint Petersburg State University (Saint Petersburg, Russia)

Alexey Masharsky

Maria Baturova

Oula Mansour

SOGAZ PROFMEDICINA LLC (Saint Petersburg, Russia)

Galina Razbitskova

Oleg Lezhnev

Larisa Gritsay

Specialized Clinical infectious Diseases Hospital (Krasnodar, Russia)

Elena Yakovchuk
